# Therapeutic efficacy and safety of Shexiang Baoxin Pill in relation to anxiety and depression in patients with coronary heart disease

**DOI:** 10.1097/MD.0000000000047605

**Published:** 2026-02-28

**Authors:** Xia Liu, Yan Zhu, Yizhen Qin, Ying Huang, Xiaodong Qian, Feng Xue, Tingbo Jiang, Mingzhu Xu

**Affiliations:** a Department of Cardiology, The First Affiliated Hospital of Soochow University, Suzhou, Jiangsu, China; b Department of Endocrinology, The First Affiliated Hospital of Soochow University, Suzhou, Jiangsu, China; c Hemodialysis Center, The First Affiliated Hospital of Soochow University, Suzhou, Jiangsu, China.

**Keywords:** anxiety, depression, prognosis, Shexiang Baoxin Pill

## Abstract

Anxiety and depression are common psychological disorders in patients with coronary heart disease (CHD). These changes might affect postoperative treatment and cardiac rehabilitation in patients with CHD. The purpose of this study was to evaluate the curative effects of the Shexiang Baoxin Pill (SBP) on anxiety and depression and prognoses in patients with CHD after percutaneous coronary intervention. A total of 236 patients with CHD and anxiety and/or depression who were admitted to our hospital from March 2021 to April 2023 were selected and non-randomly divided into a control group and a treatment group. Both groups were given medications (antiplatelets, statins, and vasodilators) and intervention therapy for CHD. The treatment group received additional treatment involving SBP administered orally (2 capsules/time, twice a day). All patients were followed up for 3 months. The basic data, laboratory examinations, symptom self-rating scale, anxiety scale (SAS)/depression scale (SDS) scores, and the incidence of cardiovascular adverse events were compared between 2 groups. Two groups of patients remained synchronous in their baseline data, laboratory data, and left ventricular systolic function. Through comparisons of the mental status of patients, we found that the SAS and SDS scores of patients in the SBP treatment group were significantly lower than those in the control group after 1 month and 3 months of treatment. The decreased value of the SAS and SDS scores in the SBP treatment group was significantly higher than the variation in the control group. The proportion of patients with anxiety or depression in the treatment group was significantly lower than the proportion before the SBP treatments. Furthermore, there was no statistically significant difference in the levels of examination data in the follow-up between the 2 groups of patients. The incidence of discomfort symptoms and adverse cardiovascular events in the treatment group was lower than in the control group. The combination of SBP with conventional treatments could not only effectively alleviate anxiety and depression and myocardial ischemia symptoms, but also safely and effectively reduce the occurrence of adverse cardiovascular events in patients with CHD. These findings provide strong clinical evidence for the treatment of dual heart disease with SBP in clinics.

## 1. Introduction

Coronary heart disease (CHD) has emerged as the leading cause of morbidity and mortality as lifestyle changes and increasingly severe aging problems have proliferated across the world.^[1,2]^ To date, despite multiple remarkable advances in interventional cardiology and modern medical treatments for CHD,^[3]^ some patients with CHD still experience symptoms such as chest tightness, chest pain, palpitations, and fatigue, and have poor prognoses. These discomfort symptoms in patients with CHD are closely related to psychological disorders, including anxiety and depression, which further exacerbate cardiac discomfort.^[4]^ It has been reported that depression and anxiety often co-occur with cardiovascular disease and are common in stable outpatients with CHD, and persistent comorbid symptoms in survivors of acute myocardial infarction (MI).^[5,6]^

Anxiety and depression are risk markers for an increased incidence of new cardiovascular diseases and a worse prognosis for existing cardiovascular diseases. Patients with CHD who have anxiety and depression are often accompanied by a greater decline in medication adherence.^[7]^ In addition, patients with CHD coinciding with mental disorders were significantly associated with an increased risk of adverse cardiovascular events compared to patients without anxiety or depression.^[8]^ These mental disorders had deleterious effects on patients’ health; thus, prevention strategies should target depression, anxiety, distress, and myocardial ischemia in patients with CHD.

Traditional Chinese medicine (TCM), an integrative medicine, has emerged as an increasingly useful and complementary approach to various diseases, such as CHD, angina pectoris, MI, and heart failure secondary to ischemic cardiomyopathy.^[9,10]^ Shexiang Baoxin Pill (SBP) is a treasured TCM formula, consisting of components including *Moschus, Calculus Bovis Sativus, Ginsenoside ginseng root, Cinnamon, Borneolum, Suhe balsam, Borneolum syntheticum, oleanolic acid acrylate,* and others.^[11]^ SBP has long been applied for the treatment of cardiovascular diseases, such as unstable angina, MI, heart failure, and myocardial ischemia.^[12–14]^ The underlying mechanisms include the promotion of angiogenesis, the amelioration of inflammation, the amelioration of cardiovascular microcirculation, improvement of endothelium dysfunction, the mitigation of dyslipidemia, repression of vascular smooth muscle cell proliferation, and the migration and restraint of cardiac remodeling.^[15–17]^

Additionally, previous studies have found that several components of SBP could protect brain neurons, regulate stress-related neurotransmitters, and induce antidepressant and antianxiety activities. Oleanolic acid acrylate can elicit antidepressant-like effects mediated by 5-HT_1_ receptors.^[18]^ The oral administration of Calculus Bovis Sativus could alleviate symptoms of neurological diseases, including hyperkinemia vascular dementia.^[19,20]^ SuHeXiang inhalation could be used as a novel therapeutic strategy for antidepressant and antianxiety effects.^[21]^ Besides, a previous study found that SBP could prevent and alleviate prolonged stress-induced depression and anxiety in mice, which might be suitable for the management of depressive and anxiety disorders in patients with cardiac diseases.^[22]^ Thus, SBP exerts outstanding effects on cardiac ischemia and psychological symptoms. Nevertheless, until recently, there has been a lack of comprehensive evidence to support the efficacy and safety of SBP combined with medical treatments for anxiety and depression in patients with CHD after percutaneous coronary intervention (PCI). This study was performed to assess whether combined SBP was associated with improved therapeutic efficacy for anxiety and depression in patients with CHD after PCI. This study may also independently provide more useful evidence that SBP has therapeutic value in terms of safety and prognoses among patients with CHD. We present this article in accordance with the CONSORT reporting checklist.

## 2. Methods

### 2.1. Study population

This was a prospective study of patients with a diagnosis of CHD who were admitted to our hospital from March 2021 to April 2023. All enrolled patients were diagnosed with CHD and followed the inclusion criteria: age ≥18 years old; diagnosed with CHD and successfully treated with PCI; confirm the presence of anxiety and depression using the assessment of symptom self-rating scale anxiety scale (SAS) and symptom self-rating scale depression scale (SDS). The SAS and SDS scales were confirmed by multiple studies with strong reliability and validity and could effectively reflect the patients’ anxiety and depression status and severity.^[23–25]^ And these patients were also followed exclusion criteria: serious heart failure (NYHA grade IV); <12-month expected survival period; patients with a previous history of mental disorders, severe depression, or suicidal tendencies; severe cerebrovascular disease in the past 6 months; malignant arrhythmias such as concomitant ventricular tachycardia, ventricular flutter, and ventricular fibrillation; malignant tumor diseases; severe and uncontrolled respiratory diseases, digestive disorders, renal diseases, hematologic disorders, infectious disease, and immunological diseases; patients abuse or reliance on substances such as morphine and alcohol in the past 6 months. Then, the 260 consecutive patients with CHD who were confirmed to have anxiety (scores between 50 and 70 using the SAS) and/or depression (scores between 50 and 70 using the SDS) were enrolled in the current study. Twenty-one patients were excluded because of incomplete data, and 3 patients were excluded due to missed follow-ups. The remaining 236 patients were enrolled in the study and were divided into a control group and a treatment group. The patients in the control group received drugs for the treatment of CHD, such as antiplatelets, statins, and vasodilators without SBP, while the patients in the treatment group were prescribed SBP (two capsules/time, twice a day, orally) in combination with drugs for CHD. All patients were followed up at 1 and 3 months after discharge, and avoided using other TCMs during follow-up sessions.

The procedures of the current study conformed to the Declaration of Helsinki with regard to ethical principles, and the participants’ data were used in accordance with the ethical standards of the institutional committees (No. 2020170). All authors confirm that each patient’s information was identified by an alias.

### 2.2. Data collection

All patients’ demographic data, clinical characteristics, heart rate, blood pressure, coronary angiographic results, and laboratory findings. Echocardiographic data, left atrial diameter, left ventricular end diastolic diameter, left ventricular end systolic diameter, left ventricular ejection fraction, and mitral flow peak velocity/early diastolic mitral annular velocity (E/e’). All enrolled subjects completed psychological assessment within 48 hours after coronary interventions, 1 month, and 3 months in the follow-up sessions. We collected the anxiety/depression symptoms evaluated by SAS and SDS scales and social restriction, the patients’ discomfort symptoms, including fatigue, palpitations, chest tightness, pain, and major cardiovascular adverse events, including readmission, revascularization, angina attacks, and heart failure attacks, during the follow-up. Experience of revascularization was identified by either PCI or coronary artery bypass grafting. Experience of angina was assessed through comprehensive questions relating to chest pain, including its anatomical distribution, precipitating and relieving factors, and duration, or any medications for relieving the development of angina pectoris, or with dynamic ST-T changes in 12-lead ECG. Experience of heart failure was assessed through symptoms of fluid retention (weight gain, edema, and shortness of breath, diuretics increase and/or intravenous diuretics), signs of heart failure, and increased N-terminal pro-B-type natriuretic peptide levels.

### 2.3. Statistical analyses

All data were analyzed using the SPSS software package (version 20.0; IBM Corp., Armonk). Continuous variables are presented in terms of mean ± standard deviation, and they were compared using 1-way ANOVA (data with normal distribution); alternatively, some are presented based on median (quartile range) values and were compared using a Kruskal–Wallis *H* test (data with non-normal distribution). Categorical variables were expressed by numbers (percentages), and then they were compared via a chi-square or Fisher exact test. A *P* < .05 (2-sided) was considered statistically significant.

## 3. Results

### 3.1. Comparison of clinical characteristics, laboratory examinations, and echocardiographic findings in patients with CHD who were confirmed to have anxiety or depression

The 236 patients were enrolled in the study and then were divided into a control group and a treatment group. The baseline characteristics, laboratory examinations, and echocardiographic findings of the populations enrolled are summarized in Table 1. The proportion of gender and NYHA grade, age, heart rate, SBP, and DBP values, level of white blood cell count, red blood cell count, hemoglobin, urea, creatinine, total bilirubin, alanine aminotransferase, aspartate aminotransferase, glucose, total cholesterol, triglycerides, low-density lipoprotein cholesterol and NT-proBNP between the 2 groups of patients did not reach statistical significance. There was no significant difference in left atrial diameter, left ventricular end diastolic diameter, left ventricular end systolic diameter, and left ventricular ejection fraction between the 2 groups. E/e’ is an important indicator of cardiac diastolic function. E/e’ value in the treatment group was significantly higher than that in the control group, indicating more serious diastolic dysfunction. These data indicate that the 2 groups of patients maintained synchrony in their baseline data and left ventricular systolic function. However, patients in the treatment group had more severe diastolic dysfunction.

**Table 1:** Baseline characteristics, laboratory findings, and echocardiographic data of study subjects.

	Control group (n = 110)	Treatment group (n = 126)	*P* value
Baseline characteristics			
Sex, male, n (%)	85 (77.27)	86 (68.259)	.122
Age, yr	61.75 ± 9.38	61.55 ± 10.64	.191
NYHA classification of cardiac function			
NYHA I, n (%)	34 (30.91)	41 (32.54)	
NYHA II, n (%)	73 (66.36)	72 (57.14)	.054
NYHA III, n (%)	3 (2.73)	13 (10.32)	
Heart rate, bpm	73.45 ± 10.56	75.63 ± 10.70	.521
SBP (mm Hg)	128.39 ± 16.75	129.17 ± 14.89	.347
DBP (mm Hg)	78.24 ± 13.46	77.72 ± 10.67	.355
Laboratory examination			
White blood cell count (×10^9^/L)	6.95 (5.80,8.71)	7.70 ± 3.13	.872
Red blood cell count (×10^12^/L)	4.54 ± 0.72	4.58 ± 0.60	.443
Hemoglobin (g/L)	138.53 ± 16.01	137.46 ± 18.47	.297
Urea (mmol/L)	5.70 ± 1.69	5.88 ± 1.65	.582
Serum creatinine (μmol/L)	70.32 ± 16.39	70.47 ± 19.08	.949
Total bilirubin (μmol/L)	12.95 (9.70,18.53)	13.40 (10.3,19.38)	.661
Alanine aminotransferase (U/L)	23.50 (16.05,79.35)	24.30 (16.00,35.80)	.863
Aspartate transaminase (U/L)	21.70 (17.18,33.70)	22.05 (17.53,35.83)	.697
Glucose (mmol/L)	5.46 (4.80,6.91)	5.65 (4.74,6.77)	.810
Total cholesterol (mmol/L)	4.03 ± 1.11	4.02 ± 1.14	.827
Triglyceride (mmol/L)	1.42 (1.10, 1.95)	1.45 (1.08, 1.99)	.944
LDL-C (mmol/L)	2.36 ± 1.05	2.29 ± 1.07	.616
NT-proBNP (pg/mL)	129.45 (47.54, 477.15)	123.65 (59.89, 599.65)	.443
Echocardiographic data			
LAd (mm)	39.59 ± 4.48	38.98 ± 6.07	.394
LVEDD (mm)	48.02 ± 5.61	48.00 (45.00, 51.00)	.777
LVESD (mm)	33.00 (30.00, 36.00)	3.00 (30.00, 36.00)	.471
LVEF (%)	59.00 (47.00, 63.00)	60.00 (46.00, 64.005)	.368
E/e’	9.22 ± 3.29	10.75 ± 4.39	.019*

DBP = diastolic blood pressure, E/e’ = mitral flow peak velocity/early diastolic mitral annular velocity, LAd = left atrial diameter, LDL-C = low-density lipoprotein cholesterol, LVEDD = left ventricular end diastolic diameter, LVEF = left ventricular ejection fraction, LVESD = left ventricular end systolic diameter, NT-proBNP = N-terminal pro-B-type natriuretic peptide, SBP = systolic blood pressure.

* *P*<.05 (control group vs treatment group).

### 3.2. Comparison of SAS/SDS scores in patients with CHD after PCI

In further comparisons of the mental status of patients, there was no statistically significant difference in SAS and SDS scores before SBP treatments between the 2 groups of patients. After 1 month of SBP treatments, the SAS and SDS scores of patients in the treatment group were significantly lower than those in the control group, which were consistent after 3 months of SBP treatments. These data indicated that SBP could alleviate anxiety and depression disorders in patients with CHD after PCI (Table 2). Then, the decreased value represents that the SAS/SDS score after 1 month and 3 months of SBP treatments, minus the SAS/SDS score before SBP treatments. The decreased value of the SAS and SDS scores in the treatment group was significantly higher than the decreased value in the control group (Table 2).

**Table 2:** Comparison of anxiety and depression status between 2 groups of subjects before SBP treatment and after SBP treatment.

	Control group (n = 110)	Treatment group (n = 126)	*P* value
Before SBP treatments			
SAS	50.00 (48.00, 53.00)	50.50 (48.00, 53.00)	.385
SDS	53.81 ± 4.52	53.60 ± 5.62	.760
After 1 mo of SBP treatments			
SAS	48.00 (45.00, 55.00)	44.00 (40.00, 46.00)	<.000*
SDS	53.00 (46.00, 56.00)	46.96 ± 6.37	<.000*
SAS†	0.55 ± 7.10	6.49 ± 5.46	<.000*
SDS†	2.35 ± 6.07	6.64 ± 6.81	<.000*
After 3 mo of SBP treatments			
SAS	43.22 ± 6.49	40.00 (36.00, 42.00)	<.000*
SDS	46.59 ± 5.76	42.5 (40.00, 46.00)	<.000*
SAS‡	6.73 ± 7.00	10.44 ± 5.49	<.000*
SDS‡	7.22 ± 6.51	11.17 ± 7.08	<.000*

SBP = Shexiang Baoxin Pill, SAS = symptom self-rating scale anxiety scale, SDS = depression scale.

* *P* < .05 (control group vs treatment group).

† The decreased value represents the SAS/SDS score after 1 month of SBP treatments minus the SAS/SDS score before SBP treatments.

‡ The decreased value represents the SAS/SDS score after 3 months of SBP treatments minus the SAS/SDS score before SBP treatments.

Based on further comparisons of the SAS and SDS scores in 2 groups of patients between before SBP treatments and after SBP treatments, in the control group, significant decreases were only detected in the SAS and SDS scores until 3 months after SBP treatments in comparison with the values before SBP treatments. Conversely, in the treatment group, significant decreases were detected in the SAS and SDS scores 1 month after SBP treatments in comparison with the values before SBP treatments, which were consistent in 3 months after SBP treatments (Table 3). These data indicated that SBP treatments could alleviate anxiety and depression disorders in patients with CHD after PCI substantially much earlier and more effectively compared to patients who did not receive SBP treatments.

**Table 3:** Comparison of anxiety and depression in 2 groups of subjects between before SBP treatments and after SBP treatments.

	Before SBP treatments	One mo after SBP treatments	Three mo after SBP treatments
SAS in control group	50.00 (48.00, 53.00)	48.00 (45.00, 55.00)	43.22 ± 6.49†
SAS in treatment group	50.50 (48.00, 53.00)	44.00 (40.00, 46.00)*	40.00 (36.00, 42.00)†
SDS in control group	53.81 ± 4.52	53.00 (46.00, 56.00)	46.59 ± 5.76†
SDS in treatment group	53.60 ± 5.62	46.96 ± 6.37*	42.5 (40.00, 46.00)†

SAS = symptom self-rating scale anxiety scale, SBP = Shexiang Baoxin Pill, SDS = depression scale.

* *P* < .05 (1 month after SBP treatments vs before SBP treatments).

† *P* < .05 (3 months after SBP treatments vs before SBP treatments).

In comparisons of the number (proportion) of patients with anxiety or depression between the 2 groups, there were no statistically significant differences in the proportion of patients with anxiety or depression between the 2 groups before SBP treatments. After 1 month of SBP treatments, the proportion of patients with anxiety or depression in the treatment group was significantly decreased compared to the proportion in the control group, and the significance were also detected after 3 months of SBP treatments (Table 4).

**Table 4:** Comparison of the number of patients with anxiety and depression between 2 groups of subjects.

	Control group (n = 110)	Treatment group (n = 126)	*P* value
Before SBP treatments			
SAS score ≥ 50, n (%)	66 (60.00%)	81 (64.29%)	.498
SDS score ≥ 50, n (%)	90 (81.82%)	95 (75.40%)	.232
After 1 mo of SBP treatments			
SAS score ≥ 50, n (%)	52 (47.27%)	13 (10.32%)	<.000*
SDS score ≥ 50, n (%)	74 (67.27%)	33 (26.19%)	<.000*
After 3 mo of SBP treatments			
SAS score ≥ 50, n (%)	21 (19.09)	2 (1.59%)	<.000*
SDS score ≥ 50, n (%)	35 (31.82%)	13 (10.32%)	<.000*
Number of cases with increased SAS or SDS scores after SBP treatments			
SAS†, n (%)	49 (44.55)	13 (10.32)	<.000*
SDS†, n (%)	17 (15.45)	4 (3.17)	.001*
SAS‡, n (%)	37 (33.64)	15 (11.90)	<.000*
SDS‡, n (%)	11 (10.00)	5 (3.97)	.066

SAS = symptom self-rating scale anxiety scale, SBP = Shexiang Baoxin Pill, SDS = depression scale.

* *P* < .05 (control group vs treatment group).

† Number of cases with increased SAS or SDS scores after 1 month of SBP treatments.

‡ Number of cases with increased SAS or SDS scores after 3 months of SBP treatments.

As we hoped, the SBP treatments would decrease the proportion of patients with anxiety or depression. We further analyzed the proportion of patients with increased SAS or SDS scores after SBP treatments in both groups. There were significant decreases in the proportion of patients with increased SAS scores after 1 and 3 months of SBP treatments between the 2 groups. And there were also remarkable decreases in the proportion of patients with increased SDS scores after 1 and 3 months of SBP treatments between the 2 groups, though the significant difference was detected after 1 month of SBP treatments (Table 4). These data indicated that SBP treatments could alleviate postoperative anxiety and depression symptoms in patients with CHD after PCI, and these psychological benefits could be observed after 1 month and 3 months of SBP treatments.

### 3.3. Comparison of laboratory examinations, discomfort symptoms, and adverse events in the follow-up sessions in patients with CHD after PCI

Adverse psychological and emotional reactions can lead to cardiovascular accidents.^[25]^ The laboratory examinations, discomfort symptoms, and adverse events in the follow-up sessions of the populations enrolled are summarized in Table 5. Three months after the treatments, all enrolled patients had completed blood routine and other biochemical tests. There was no statistically significant difference in white blood cell count, red blood cell count, platelet count, urea, creatinine, total bilirubin, alanine aminotransferase, aspartate aminotransferase, fasting blood glucose, total cholesterol, and triglycerides between the 2 groups of patients. Further comparison of the discomfort symptoms of 2 groups of patients, we found that the proportion of patients with fatigue, palpitations, chest tightness, chest pain, and social restriction in the treatment group was lower than that in the control group. Moreover, the proportion of patients with chest tightness and chest pain, and the proportion of patients with social restriction in the treatment group were significantly lower than in the control group. In addition, by comparing the incidence of adverse events, the incidence of readmission, revascularization, angina attacks, and heart failure attacks was lower in the treatment group than in the control group. Moreover, there were significant decreases in the incidence of readmission and angina attacks compared to the control group.

**Table 5:** Comparison of laboratory examinations, discomfort symptoms, and cardiovascular adverse events in the follow-up between the 2 groups.

	Control group (n = 110)	Treatment group (n = 126)	*P* value
Laboratory examinations			
White blood cell count (×10^9^/L)	7.29 (5.91, 8.76)	6.71 ± 2.08	.083
Red blood cell count (×10^12^/L)	4.72 ± 0.70	4.52 ± 0.56	.170
Platelet count (×10^9^/L)	205.67 ± 60.60	217.20 ± 93.94	.601
Urea (mmol/L)	5.70 ± 1.69	5.88 ± 1.65	.582
Serum creatinine (μmol/L)	73.88 ± 18.93	74.30 (63.43, 88.40)	.687
Total bilirubin (μmol/L)	13.37 (10.70, 16.90)	12.35 (10.12, 16.78)	.443
Alanine aminotransferase (U/L)	32.31 ± 19.48	26.05 (18.30, 39.83)	.391
Aspartate transaminase (U/L)	22.40 (17.60, 27.20)	21.50 (18.25, 25.95)	.553
Glucose (mmol/L)	6.13 (5.26, 7.27)	6.14 ± 1.36	.230
Total cholesterol (mmol/L)	3.05 ± 0.82	3.13 ± 1.13	.972
Triglyceride (mmol/L)	1.31 (1.05, 1.64)	1.59 ± 0.77	.344
Number of cases with discomforts			
Fatigue, n (%)	26 (23.64)	18 (14.29)	.066
Palpitation, n (%)	23 (20.91)	19 (15.08)	.243
Chest tightness and pain, n (%)	31 (28.18)	20 (15.87)	.022*
Social restrictions, n (%)	30 (27.27)	19 (15.08)	.021*
Number of cases with cardiovascular adverse events			
Readmission, n (%)	14 (12.73)	5 (3.97)	.018*
Coronary revascularization, n (%)	10 (9.09)	4 (3.17)	.068
Angina pectoris attack, n (%)	14 (12.73)	6 (4.76)	.034*
Heart failure attack, n (%)	15 (13.64)	18 (14.29)	.886

* *P* < .05 (control group vs treatment group).

## 4. Discussion and conclusions

CHD is a common cardiovascular disease with high mortality, which is closely related to psychological factors. Anxiety and depression are common psychological disorders in patients with cardiovascular disease, which are easily confused with symptoms of cardiovascular disease. In addition, long-term stress, unhealthy lifestyles, and negative emotions could increase the occurrence of adverse cardiovascular events and accelerate arteriosclerosis.^[25]^ Therefore, treatments for patients with CHD should treat symptoms of anxiety and depression simultaneously, which can help promote patients’ recovery. In the current study, we, for the 1st time, performed a prospective analysis of the underlying cardioprotective effects and psychological effects of SBP based on the clinical perspectives, and found that SBP could alleviate anxiety and depression disorders, reduce the occurrence of discomfort symptoms, and improve the incidence of adverse events in the early postoperative stage among patients with CHD after PCI. The safety of SBP was also confirmed in these patients.

SBP, a formulated Chinese herbal mixture, might have therapeutic effects for several reasons. On the 1 hand, SBP has antithrombotic effects, including dilated blood vessels, increased myocardial resistance to ischemia and hypoxia, and therapeutic effects on CHD and angina pectoris.^[26,27]^ On the other hand, various components of SBP directly regulate neurotransmitters. Musk and bezoar can protect brain neurons from hypoxic damage.^[28]^ Ginsenoside and oleanolic acid could regulate neurotransmitters such as aminobutyric acid, dopamine, and 5-hydroxytryptamine, exhibiting antidepressant and antianxiety activity.^[18,29]^ Therefore, SBP has synergistic effects of reducing anxiety, tension, and depression. SBP may be particularly suitable for the management of depressive and anxiety disorders in patients with cardiac conditions. In a previous study, mice underwent 6 weeks of chronic unpredictable mild stress to induce depression- and anxiety-like behavior, and treatment with SBP was able to stress-induced reductions of norepinephrine and dopamine metabolites and the expression levels of brain-derivedneurotrophic factor, nerve growth factor, and glial cell-derived neurotrophic factor in related brain regions.^[22]^ It therefore appeared that SBP was particularly suited to preventing and alleviating stress-induced mild mood and anxiety disorders.

In the current study, we investigated the effect of SBP on anxiety and depression in patients who were diagnosed with CHD after PCI. We found that SBP treatment could significantly lower SAS/SDS scores and lower the proportion of anxiety and depression patients. In addition, some CHD patients displayed an increase in anxiety and depression after PCI in the follow-up. In the current study, SBP treatments significantly lowered the proportion of patients with increasing instead of decreasing SAS and SDS scores. These results confirmed that the combined use of SBP could effectively reduce the incidence and severity of anxiety and depression psychological disorders in patients with CHD, and also alleviate anxiety and depression emotional disorders in early postoperative patients. Thus, the application of SBP may be particularly suitable for the management of depressive and anxiety disorders in patients with cardiac conditions.

Anxiety and depression are not only prevalent in patients with CHD but also can predict these patients’ cardiovascular events. Active intervention or treatment of anxiety and depression can improve patient symptoms and long-term adverse cardiovascular events in patients with CHD.^[30]^ Excessive excitement or excessive worry and anxiety can trigger physical disorders, leading to poor prognosis in patients with CHD. To seek the improvement of SBP, we explored the safety and the incidence of cardiovascular adverse events in this study. We found that SBP treatment did not increase the burden on the liver and kidneys, and had no adverse effects on blood lipids. Besides, SBP could alleviate patients’ discomfort symptoms, reduce the incidence of cardiovascular adverse events, and improve the prognosis of patients with CHD. Thus, SBP might have a “dual heart” mechanism, as it not only dilates blood vessels to improve myocardial ischemia but also reduces anxiety, tension, and depression. Some limitations inherent to the study should be acknowledged. The enrolled subjects were limited. The study is a 3-month short-term research.

## 5. Conclusions

The treatment of CHD patients should account for both the treatment of CHD and the intervention of anxiety and depression symptoms. In this study, we, for the 1st time, have found that SBP had preventive activity in relation to antidepressant and anxiolytic effects and improved general symptoms and cardiovascular adverse events. It therefore appears that SBP is particularly suited to preventing ischemic attack and alleviating mild anxiety and depression disorders. Therefore, the early assessment of coronary artery disease and anxiety and depression, as well as the early implementation of personalized intervention and comprehensive health management plans, may improve the prognosis of patients with CHD. This clinical practice may help clinicians to improve myocardial ischemia and reduce anxiety, tension, and depression based on the SBP treatments.

## Acknowledgments

This study was supported by the grant of the Chinese Association of Integrated Traditional Chinese and Western Medicine-Hehuang Research Foundation (No. HMP2005005P).

## Author contributions

**Data curation:** Xia Liu, Yan Zhu, Yizhen Qin.

**Project administration:** Xia Liu, Mingzhu Xu.

**Investigation:** Yan Zhu.

**Software:** Yizhen Qin, Xiaodong Qian, Mingzhu Xu.

**Visualization:** Yizhen Qin.

**Formal analysis:** Ying Huang, Feng Xue.

**Validation:** Ying Huang.

**Conceptualization:** Xiaodong Qian, Mingzhu Xu.

**Supervision:** Xiaodong Qian, Tingbo Jiang.

**Writing – review & editing:** Feng Xue, Tingbo Jiang, Mingzhu Xu.

**Writing – original draft:** Mingzhu Xu.
